# Efficacy of the perfluorocarbon dodecafluoropentane as an adjunct to pre-hospital resuscitation

**DOI:** 10.1371/journal.pone.0207197

**Published:** 2018-11-29

**Authors:** Alicia M. Bonanno, Todd L. Graham, Lauren N. Wilson, Brianne M. Madtson, James D. Ross

**Affiliations:** 1 Department of Surgery, Oregon Health and Science University, Portland, Oregon, United States of America; 2 Division of Trauma and Acute Care Surgery, Oregon Health and Science University, Portland, Oregon, United States of America; University Hospital Hamburg Eppendorf, GERMANY

## Abstract

**Background:**

Hemorrhage is the most common cause of preventable death in the pre-hospital phase in trauma, with a critical capability gap optimizing pre-hospital resuscitation in austere environments. One promising avenue is the concept of a multi-functional resuscitation fluid (MRF) that contains a blood product backbone with agents that promote clotting and enhance oxygen delivery. Oxygen therapeutics, such as hemoglobin based oxygen carriers(HBOCs) and perfluorocarbons(PFCs), may be a critical MRF component. Our purpose was to assess the efficacy of resuscitation with a PFC, dodecafluoropentane(DDFPe), compared to fresh whole blood(FWB).

**Methods and findings:**

Forty-five swine(78±5kg) underwent splenectomy and controlled hemorrhage via femoral arterial catheter until shock physiology(lactate = 7.0) was achieved prior to randomization into the following groups: 1) Control-no intervention, 2)Hextend-500mL, 3)FFP-500mL, 4)FFP+DDFPe-500mL, 5)FWB-500mL. Animals were observed for an additional 180 minutes following randomization.

**Results:**

Baseline physiologic values did not statistically differ. At T = 60min, FWB had significantly decreased lactate(p = 0.001) and DDFPe was not statistically different from control. There was no statistical significance in tissue oxygenation(StO2) between groups at T = 60min. Survival was highest in the FWB and Hextend groups(30% at 180min). Kaplan-Meier analysis showed decreased survival of DDFPe+FFP in comparison to FWB(p<0.05) and was not significantly different from control or FFP. Four animals who received DDFPe died within 10 minutes of administration. This study was limited by a group receiving DDFPe alone, however this would not be feasible in this lethal swine model as DDFPe given its small volume.

**Conclusion:**

DDFPe administration with FFP does not improve survival or enhance tissue oxygenation. However, given similar survival rates of Hextend and FWB, there is evidence that an ideal MRF should contain an element of volume expansion to enhance oxygen delivery.

## Introduction

Poor availability of blood products in austere environments is a challenge to effective pre-hospital trauma resuscitation. Therefore, there is a critical need for the development of a multi-functional resuscitation fluid (MRF), which would act in lieu of blood component therapy or fresh whole blood without the complex logistical tail. An optimal MRF will likely include a blood product based backbone, agents that promote clotting, and oxygen therapeutics (OT) to enhance oxygen carrying capacity.

Oxygen therapeutics, such as hemoglobin based oxygen carriers (HBOC) or perfluorocarbons (PFC), have shown promise as an alternative for resuscitation and oxygen delivery to tissues.[1,2] However in recent trials, some have proven to carry significant risk to specific patient populations eliciting myocardial infarction, systemic vasoconstriction and renal dysfunction.[3] Despite the controversial interpretation of these studies, continued development of OTs has resulted in new formulations aimed at preventing interaction with vasculature, reducing extravasation, and decreasing oxidative stress.[2]

Perfluorocarbons are organofluorines (C_x_F_x_) which have the ability to dissolve and transport oxygen.[1,2] Liquid PFCs have a low surface tension, are chemically and metabolically inert, and are eliminated by evaporation all which contribute to their function as an effective OT.[1] Dodecafluoropentane emulsion (DDFPe), is a PFC that as a gas at body temperature can transport more oxygen per weight than liquid PFCs.[4] DDFPe can be administered as a single small dose with rapid clearance and minimal residuals or side effects.[5] It has shown promise in animal models, including rabbit ischemic stroke studies that had a protective effect by decreasing infarct volume.[6,7,8] Specifically, Culp et al demonstrated median percent infarct volume (p = 0.001) for all rabbits treated with DDFPe (0.30%) compared with controls (3.20%).[6] Additionally, Strom et al used a mouse model with myocardial ischemia, in which mice treated with DDFPe had a reduction in infarct size by approximately 72% in 24 hours (p<0.01).[9]

In the context of MRFs, DDFPe in combination with a blood product backbone like lyophilized or spray dried plasma, may be a beneficial agent that is immediately available, shelf stable, easily transportable, and have resuscitative and hemostatic properties.[10,11,12] This resuscitation fluid would theoretically achieve both effective oxygen delivery and hemorrhage control, while also decreasing oxidative stress. The purpose of this study was to determine whether resuscitation with DDFPe in plasma would restore oxygen delivery to end organs more effectively than resuscitation with plasma alone, as evidenced by survival and indices of tissue oxygen delivery. Also, as FWB is the gold standard for resuscitation in order to reverse coagulopathy during hemorrhagic shock, we examined the impact of DDFPe in plasma on coagulation in comparison to blood product resuscitation, as evidenced by viscoelastic measures of thrombus formation and degradation.

## Materials and methods

Institutional Animal Care and Use Committee Approval for this study was obtained and experiments were carried out in a facility accredited by the Association for Assessment and Accreditation of Laboratory Animal Care at Oregon Health and Science University, Portland, Oregon. Animals were obtained from Oak Hill Genetics, animals were used in accordance with *The Guide for the Care and Use of Laboratory Animals*.

Male Yorkshire swine (78±5kg) were sedated with Telazol (IM) injection and maintained under a surgical plane of anesthesia for the entire duration of the experiment with Isoflurane after oro-tracheal intubation. Vascular access was achieved by percutaneous technique or cut-down. Lines placed included right carotid flow probe monitor, right external jugular pulmonary artery (PA) catheter, left carotid arterial line for monitoring, a left external jugular venous line for fluid administration, and a femoral arterial line for hemorrhage. Animals were then monitored using both high fidelity (>500Hz) and low fidelity (Q1min) telemetry including EKG, invasive arterial pressures, cardiac output, systemic vascular resistance, EtCO_2_, SVO_2_, VO_2_, near-infrared spectroscopy was measured utilizing Medtronic’s Invos 5100 sensors placed on the left pectoralis and left abductor brevis (STO_2_), and carotid flow.

**Fig 1:**
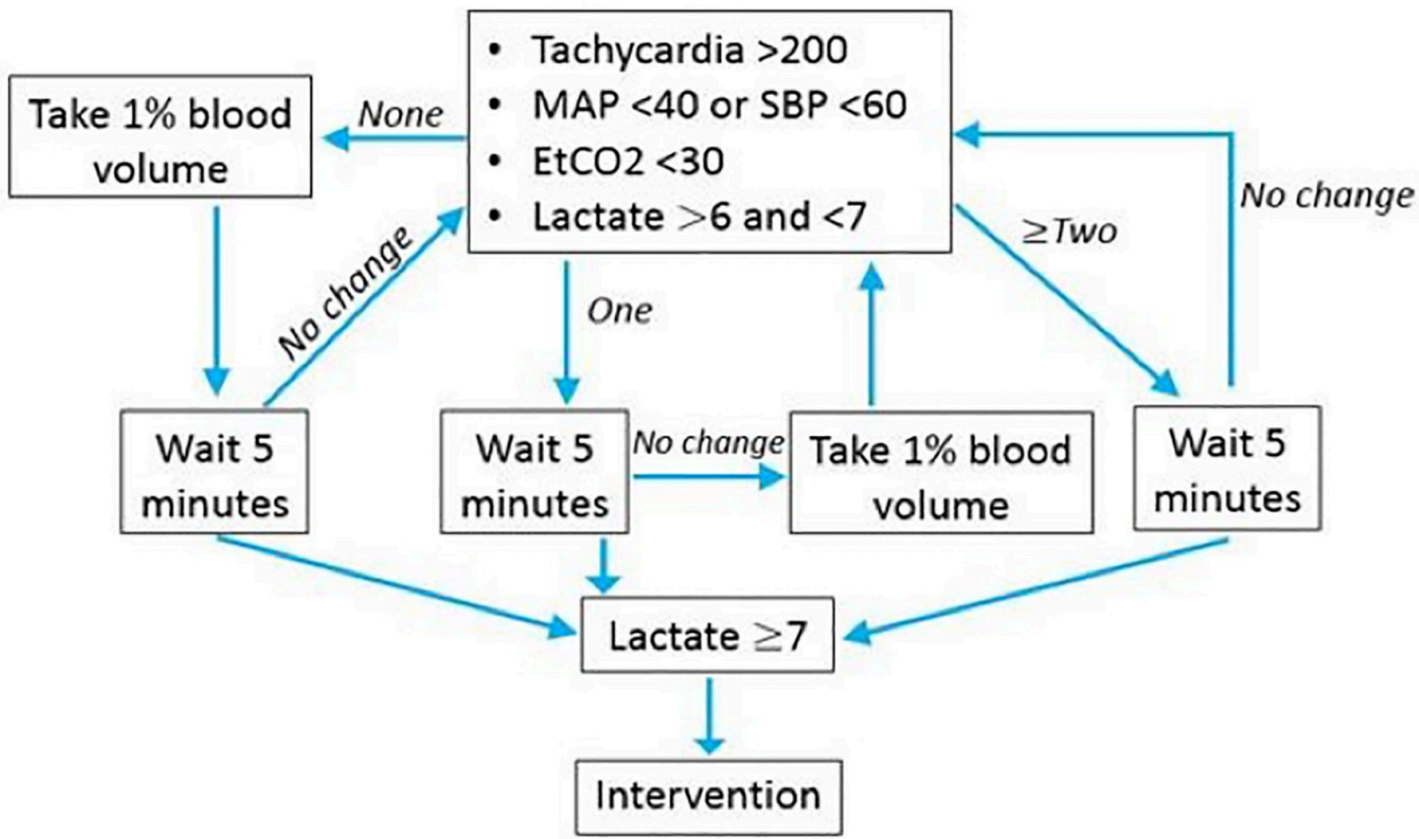
Hemorrhage algorithm. Algorithm utilized following hemorrhage of 35% blood volume to achieve lactate of 7 and hemodynamic instability.

Laparotomy and splenectomy were performed to eliminate auto-transfusion and to include a standardized degree of soft tissue injury. Suprapubic catheter was placed for urine output monitoring and the abdomen was closed. Animals were pre-treated with ketoralac, to prevent the potential for pulmonary anaphylaxis due to micro particle interaction. Following a ten-minute stabilization period, controlled hemorrhage was initiated via femoral arterial catheter and designated H = 0. Thirty-five percent of blood volume was removed based on a calculation of 65mL/kg using a peristaltic pump for accurate volume and rate monitoring over thirty minutes. At H = 45, if the animal’s lactate reached a threshold of ≥7.0 the resuscitation would begin. If the animal did not reach this threshold, depending on their MAP, EtCO_2_ and HR, an additional 1% blood volume would be removed sequentially using the algorithm in Fig 1. The animals were then randomized into one of five groups: 1) Control–no intervention (n = 5), 2) Hextend, 500mL (n = 10), 3) FFP, 500mL (n = 10), 4) FFP+DDFPe (DDFPe), 500mL (n = 10), 5) FWB, 500mL (n = 10). The volume of 500mL for initial resuscitation was chosen based off of previously developed models of hemorrhagic shock. Subsequent to randomization, the commencement of resuscitation was denoted as T = 0. Group 2–5 resuscitation was administered via a Belmont Rapid Transfuser at a rate of 100mL/min. Group 4 received a co-administration of DDFPe (IV push) at 2mL/min.

Animals were observed and telemetry collected for 180 minutes to stimulate an extended pre-hospital resuscitation period. At the completion of the experiment, animals were euthanized with IV Somnasol (0.22mL/kg) and underwent necropsy for gross pathology with samples collected for future histologic examination.

### Data collection and study endpoints

Arterial blood samples were taken prior to hemorrhage (baseline), Intervention (T = 0), T = 60, and T = 180 or at the time of death. Arterial blood gas samples were collected at Q15min. Data collected for primary endpoints included survival, indices of cardiovascular and cardiopulmonary function, indices of tissue oxygenation, blood gas and chemistry. Secondary endpoints included clotting and coagulation status and histopathological examination of lung tissue for eosinophil infiltration and evidence of anaphylaxis.

### Statistical analysis

Baseline data points were compared using ANOVA. Clinical and laboratory values were measured and analyzed by ANOVA with post-hoc pairwise comparisons where appropriate (Holm-Sidak). Kaplan-Meier survival analysis was performed.

## Results

### Baseline animal characteristics

There were no statistical differences in baseline characteristics including weight (78±5kg) and percentage of hemorrhage (37.7±3.0%). Baseline lactate was also similar between groups. Preparation time did not differ amongst groups. There was no significant difference in lactate, pH, blood counts and chemistries or viscoelastic parameters of coagulation post-hemorrhage.

### Physiologic measures 60 minutes post-intervention (Table 1)

**Table 1:** Physiologic parameters at 60-minutes.

*Variable*	*CON (n = 3)*	*HEX (n = 7)*	*FFP (n = 10)*	*DDFPe (n = 6)*	*FWB (n = 9)*	*p-value*
Heart rate, beats/min	149 ± 30	186 ± 23	174 ± 41	179 ± 33	197 ± 24	0.222
Carotid SBP, mmHg	58 ± 12	80 ± 18	67 ± 19	59 ± 15	77 ± 13	0.081
Pulmonary SBP, mmHg	26 ± 2	26 ± 4	23 ± 4	22 ± 8	26 ± 5	0.467
CVP, mmHg	6 ± 1	7 ± 1	5 ± 1	6 ± 2	4 ± 2*	0.033
CO, L/min	3.0 ± 1.4*	5.4 ± 0.9	3.7 ± 0.8*	3.4 ± 0.9*	4.5 ± 1.0	<0.001
SVR, dyn·s/cm	632 ± 231	610 ± 111	825 ± 437	665 ± 272	732 ± 138	0.582
EtCO2, mmHg	30 ± 6‡*	41 ± 5	37 ± 6‡	35 ± 7‡	45 ± 3	<0.001
Hgb, g/dL	8.5 ± 0.9	7.4 ± 0.5‡	8.2 ± 0.9‡	8.1 ± 0.8‡	9.4 ± 0.7	<0.001
Potassium, mmol/L	6.2 ± 0.9	4.7 ± 0.6†	4.8 ± 0.6†	5.4 ± 0.7	4.6 ± 0.4†	0.002
Lactate, mmol/L	15.7 ± 2.0	8.7 ± 3.4†	10.0 ± 2.4†	10.7 ± 2.8	7.4 ± 2.5†	0.001
DO2	337 ± 153	545 ± 70†	406 ± 64‡*	366 ± 89‡*	549 ± 111†	<0.001

Note:

^†^ Symbolizes a statistically significant differnce to CON group

^‡^ Symbolizes a statistically significant difference to FWB group

*Symbolizes a statistically signifcant difference from HEX

Cardiac output was noted to be significantly lower in control (3.0±1.4L/min), FFP (3.7±0.8L/min), and DDFPe (3.4±0.9L/min) in comparison to Hextend (5.4±0.9L/min), p<0.001. End tidal CO_2_ was significantly lower in control (30±6mmHg), FFP (37±6mmHg), and DDFPe (35±7mmHg) when compared to FWB (45±3mmHg), p<0.001. As predicted, FFP, DDFPe and Hextend had decreased hemoglobin levels in comparison to FWB, p<0.001. Potassium values were decreased in FFP (4.8±0.4mmol/L), FWB (4.6±0.4mmol/L) and Hextend (4.7±0.6mmol/L) compared to control (6.2±0.9mmol/L), p = 0.002. However, there was no significant difference in potassium values between control and DDFPe.

There were no significant differences in tissue oxygenation (StO_2_) or SvO_2_ amongst groups. DO_2_ was significantly decreased in DDFPe (366±89 mL/min) in comparison to FWB (549±111 mL/min) and Hextend (545±70 mL/min), p<0.001. There was no significant difference in DO_2_ between DDFPe versus Control and FFP (Table 1).

### Metabolic burden and coagulopathy

There was no significant difference in pH at T = 60. However, there were significant differences in lactate levels amongst groups. Lactate in the control group was significantly higher than FWB (p<0.001), Hextend (p = 0.007), and FFP (p = 0.027, Table 1). There was no statistically significant difference detected between lactate levels for control and DDFPe groups. All other group comparisons of lactate were not found to be statistically significant.

There were no significant differences in Clotting Time, Clot Formation Time, Maximum Clot Formation, alpha angle, or lysis 30 at baseline and T = 60 (Table 2).

**Table 2:** Viscoelastic properties of coagulation.

*Variable*	*CON (n = 3)*	*HEX (n = 4)*	*FFP (n = 4)*	*DDFPe (n = 3)*	*FWB (n = 4)*	*p-value*
Clotting time, seconds	660 ± 118	493 ± 134	618 ± 129	665 ± 94	590 ± 39	0.260
Clot formation time, seconds	213 ± 99	130 ± 59	127 ± 42	230 ± 108	145 ± 22	0.206
Maximum clot firmness, mm	64 ± 6	60 ± 8	69 ± 2	58 ± 5	64 ± 8	0.254
α angle, degrees	55 ± 12	66 ± 9	67 ± 7	53 ± 13	64 ± 3	0.198
Lysis 30, %	100.0 ± 0.0	99.3 ± 1.5	99.5 ± 0.6	99.0 ± 1.7	98.8 ± 1.0	0.661

### Survival analysis

**Fig 2:**
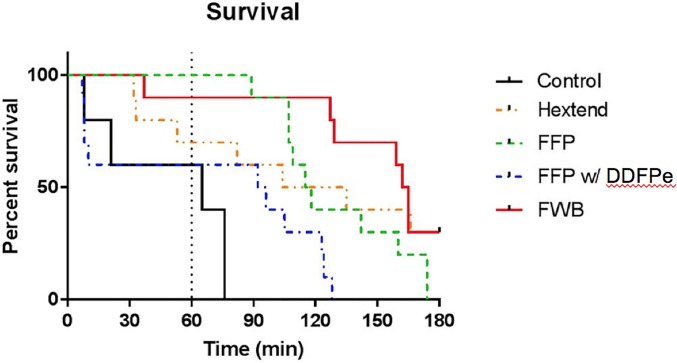
Survival analysis. Kaplan-Meier survival curve at 180 minutes post intervention. The vertical dotted line represents 60 minutes post intervention. Physiologic analysis was performed at this time point.

There were six early deaths from exsanguination that occurred prior to intervention and these animals were excluded from the study. Four animals who received FFP with DDFPe expired within 10 minutes following administration (Fig 2). Only one control animal died within 10 minutes. Fresh Whole Blood and Hextend had the greatest survival with 30% in each group living to the end of the study period. Comparisons between each group was performed at T = 180, FFP and FWB were found to have statistically significant improved survival in comparison to control (p<0.001 and p = 0.005 respectively). DDFPe was found to have decreased survival in comparison to FWB (p<0.001) and there was no statistically significant difference in survival in comparison to control (p = 0.361). All other intergroup comparisons were not significant.

## Discussion

This study demonstrates that DDFPe administration with FFP does not improve survival or enhance tissue oxygenation in this swine hemorrhagic model. However, we observed the importance of volume expansion as an element in MRFs as evidenced by similar survival of FWB and Hextend animals. Animals who received DDFPe had similar outcomes to the control group, although, several factors may contribute to this, including compliment activation-related psuedoallergy (CARPA) or the use of FFP as a carrier fluid.

The absence of data to support DDFPe efficacy after administration may not be definitively attributed to a lack of ability to carry oxygen to tissues as swine are known to exhibit symptoms of CARPA in response to liposome-induced C activation, various nanoparticles, and other particulate supramolecular structures.[12,13,14] As the intravenous distribution time of DDFPe is 1.1±0.6 min with an elimination half-life of 14±11min, CARPA may have been a factor in early deaths occurring within the first 10 minutes following administration.[15] However, when observing these particular deaths, we did not identify statistically significant elevations in PA pressures or reduction in EtCO_2_ as expected in a CARPA-like response to infusion,[16] nor did histopathology identify any eosinophilic infiltration. With these observations of unchanged PA pressures and EtCO_2_, we can also infer that animals did not experience a transfusion reaction to the carrier fluid (FFP).

The gold standard for trauma resuscitation is 1:1:1 transfusion.[17] However, we observed equivalent survival outcomes with volume matched Hextend and FWB in a 3-hour pre-hospital period where definitive hemorrhage control is assumed. This effect can be explained by comparable tissue oxygen delivery between Hextend and FWB as evidenced by similar DO_2_ levels. This is strikingly similar to the results described by Sheppard et al. who showed equivalency in resuscitative effects of Hextend and FWB in non-human primates.[18] However in the context of pre-hospital combat casualty care, definitive hemorrhage control may not be achieved and therefore continued resuscitation with volume expanders that lack promoters of coagulation may have limited use due to exacerbation of hemodilution.[17] From this, we can extrapolate that an ideal MRF will contain a volume expander in addition to varying promoters of hemostasis to attempt to replicate FWB transfusion.

The concept of prioritizing volume expansion over oxygen carrying capacity is feasible in pre-hospital care as demonstrated by Spinella et al. and the PROMMTT trial which have reported hospital arrival hemoglobin levels above a clinically critical hemoglobin of 7 (9.0±2.6g/dl and 11.7±3.21g/dl, respectively) in the military and civilian trauma populations.[19,20] This, however, necessitates the need for the addition of procoagulants (e.x. FFP, platelets, PCC, fibrinogen) in prolonged field care to avoid dilutional coagulopathy and worsening of the lethal triad.[18] Although we did not demonstrate differences in our coagulation parameters amongst groups, this is likely due to the fact that this study was powered for survival as opposed to coagulation and it has been well established that clot promotion is critical in trauma resuscitation.[18,19]

The present study is limited by the use of a controlled hemorrhage model, instead of an injury model with uncontrolled hemorrhage. The investigators purposely chose controlled hemorrhage to ensure hemodynamic and physiologic uniformity for comparison. In addition, we developed an algorithm for controlled hemorrhage that used metabolic status as an outcome as opposed to previous volume or pressure models.[21] Other investigators have suggested the use of a concept referred to as “controlled uncontrolled hemorrhage” where an injury model is used to achieve hemorrhagic shock where the end state of that shock can be maintained by arbitrary hemorrhage control.[21] However, we feel that this approach is equally limited in use by the lack of a metabolic endpoint for investigation of oxygen therapeutics specifically as that model had a different lactate and base deficit at baseline and end of shock periods.[22] Another limitation of this study is the lack of a DDFPe infusion group in the absence of FFP. While we considered this in the experimental design, it was discarded due to the fact that the model employed in this study was predicted as lethal without a significant volume resuscitation component and therapeutic DDFPe alone consisted solely of 10mL of infusion.

## Conclusion

FFP with DDFPe did not increase overall survival within this controlled hemorrhagic swine model. While this study did not show enhanced oxygen delivery during severe hemorrhagic shock with DDFPe, we did demonstrate a similar DO2 between Hextend and FWB. This does not necessarily preclude the use of an OT in different formulations of a multi-functional blood substitute in that prolonged field care without definitive hemorrhage control may result in critical hemodilation requiring additional oxygen carrying capacity.

## Supporting information

S1 Checklist NC3Rs arrive guidelines checklist (fillable).pdf. (PDF)
